# Does attentional suppression occur at the level of perception or decision-making? Evidence from Gaspelin et al.’s (2015) probe letter task

**DOI:** 10.1007/s00426-022-01734-3

**Published:** 2022-09-12

**Authors:** Dirk Kerzel, Olivier Renaud

**Affiliations:** Department of Psychology, Faculté de Psychologie et des Sciences de L’Éducation, Université de Genève, 40 Boulevard du Pont d’Arve, 1205 Geneva, Switzerland

## Abstract

Visual attention is often inadvertently captured by salient stimuli. It was suggested that it is possible to prevent attentional capture in some search tasks by suppressing salient stimuli below baseline. Evidence for attentional suppression comes from a probe task that was interleaved with the main search task. In the probe task of Gaspelin et al. (Psychol Sci 26(11):1740–1750, 2015. 10.1177/0956797615597913), letters were shown on the stimuli of the search display and participants had to identify as many letters as possible. Performance was found to be worse for letters shown on the distractor compared to non-salient non-target stimuli, suggesting that distractor processing was suppressed below baseline. However, it is unclear whether suppression occurred at the level of perception or decision-making because participants may have reported letters on the distractor less frequently than letters on nontargets. This decision-level bias may have degraded performance for letters on distractor compared to nontarget stimuli without changing perception. After replicating the original findings, we conducted two experiments where we avoided report bias by cueing only a single letter for report. We found that the difference between distractor and nontarget stimuli was strongly reduced, suggesting that decision-level processes contribute to attentional suppression. In contrast, the difference between target and non-target stimuli was unchanged, suggesting that it reflected perceptual-level enhancement of the target stimuli.

**Supplementary Information:**

The online version contains supplementary material available at 10.1007/s00426-022-01734-3.

## Introduction

From the many stimuli in the environment, only a few can be selected for in-depth processing. A number of theories have been advanced to explain how selection is achieved (Bundesen, 1990; Desimone & Duncan, 1995; Eimer, 2014; Schneider, 2013; Treisman & Gelade, 1980; Wolfe, 2021). An interesting test case for any theory of visual attention is a situation with a highly salient distractor competing with a less salient target because it reveals the interplay of bottom-up saliency and top-down search goals (Awh et al., 2012; Kruger et al., 2017; Lamy et al., 2012; Luck et al., 2021).

To explain how top-down search goals can prevent distraction, Gaspelin and Luck (2018b) proposed that processing of salient-but-irrelevant distractors is suppressed below baseline level. They define attention broadly as “a set of processes by which some stimuli receive greater processing resources or greater weight in decisions at the expense of others” (p. 80). However, it is also possible to consider attention exclusively as a resource in the context of perceptual processing. In particular, attention may result in “spatially specific processing enhancements for candidate target objects at specific locations” (Eimer, 2014 p. 526). In the current contribution, we distinguish between early attentional processes that modulate perception (Carrasco, 2011; Dosher & Lu, 2000) and later attentional processes that modulate decision-making (Luck & Thomas, 1999; Schönhammer et al., 2017; Shiu & Pashler, 1994). That is, we suggest that suppression may occur at early and late stages, an assumption that was already implicit in Gaspelin and Luck’s (2018b) definition of attention. Perceptual-level suppression is consistent with the early occurrence of an electrophysiological marker of attentional suppression, the P_D_ component. The latency of the P_D_ is typically around 200–250 ms after stimulus onset (Burra & Kerzel, 2013; Gaspar & McDonald, 2014; Hickey et al., 2009), but some studies reported latencies as short as 110–140 ms (Sawaki & Luck, 2010; Weaver et al., 2017). In contrast, changes in the weight of stimuli in decision processes occur after early perceptual processes. Thus, we refine the idea of attentional suppression introduced by Gaspelin and Luck (2018b) by distinguishing between perceptual- and decision-level suppression. Among other things, perceptual-level suppression may reduce the subjective clarity of the stimulus, whereas decision-level suppression may reduce the probability of reporting a stimulus.

**Fig. 1:**
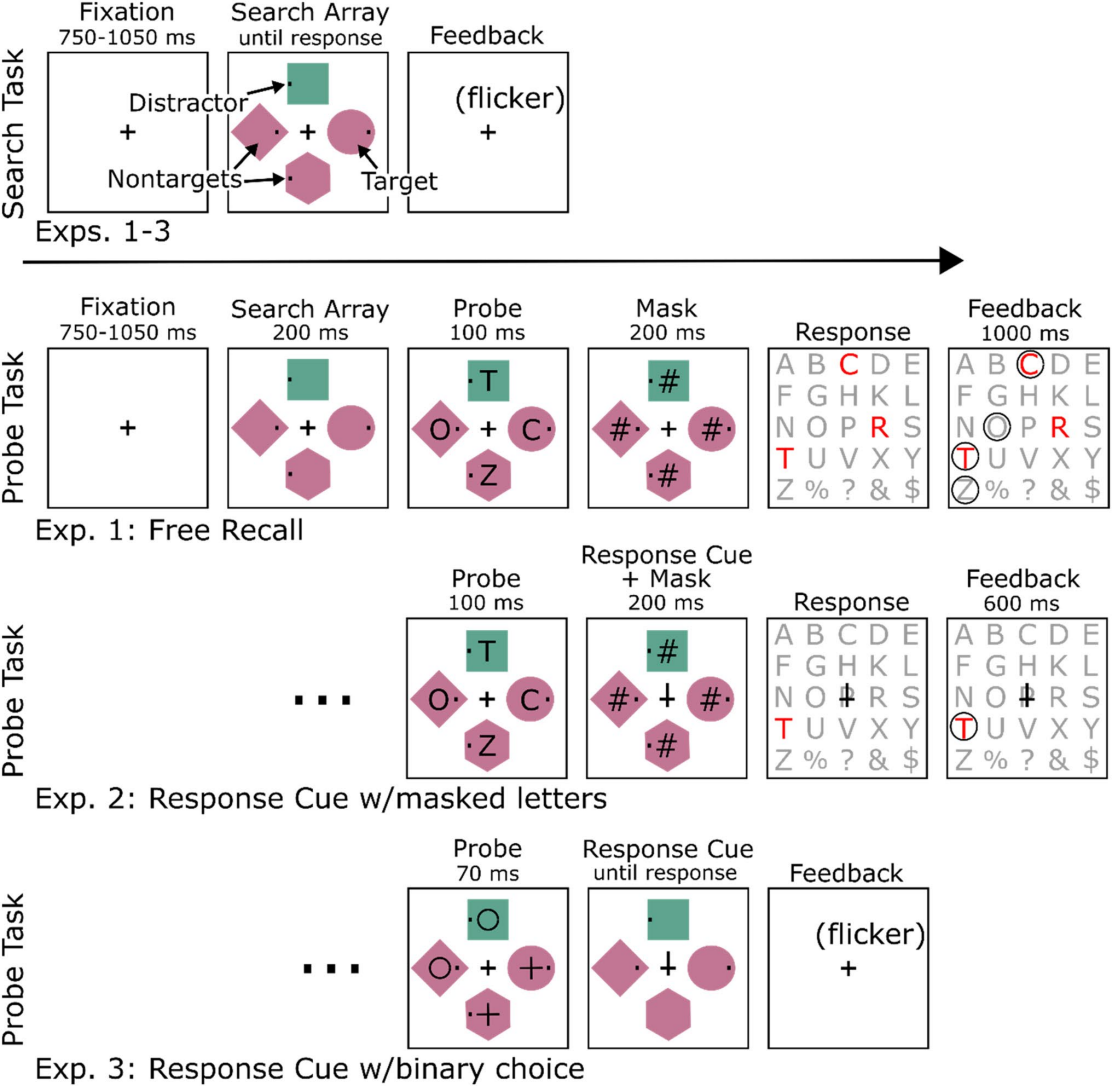
Schematic illustration of the search and probe paradigms in Experiments 1–3. Participants were instructed to search for a specific shape (e.g., the circle). On search trials, participants indicated whether the dot inside the target shape was on the left or right. In the interleaved and less frequent probe task, participants indicated the identity of letters that were briefly flashed on the search array. In the free recall procedure, participants reported as many letters as possible. In the procedure with response cue, participants reported the letter indicated by the response cue

While the suppression of salient-but-irrelevant distractors is desirable, the ability to successfully deal with distractors is limited. One condition promoting attentional suppression is feature search (Gaspelin et al., 2015). In feature search, participants search for a particular shape among various shapes and report a feature inside this shape (Bacon & Egeth, 1994). For instance, participants may search for a circle among a diamond, a square and a hexagon (see Fig. 1), and report the position of a dot inside the circle. In these small search displays with various shapes, RTs were found to be shorter when one of the three non-targets had a color different from the others. The stimulus with the odd color was irrelevant because the target shape was always shown in the majority color. Typically, RTs increase when one of the nontarget shapes has a color different from the others (Theeuwes, 1991, 1992), but in this condition, search times were shorter in the presence of a distractor. To explain the reduced search times, the suppression hypothesis assumes that processing of the distractor was pushed below baseline level. As a result, the effective set size of the search display dropped from four to three stimuli, which allowed for shorter search times. Similarly, oculomotor capture by the distractor was reduced compared with nontargets (Adams et al., 2022; Gaspelin et al., 2017). That is, when the size of the feature inside the target required participants to make a saccade to the target, fewer erroneous saccades went to the distractor than to nontargets. However, it is unclear whether perceptual- or decision-level suppression accounts for these findings. Because the distractor was never the target, it could be entirely excluded from decisions about where to direct the saccade. Alternatively, the lack of eye movements to the distractor could mean that the stimulus was perceived less clearly.

Thus, search RTs and oculomotor capture do not allow for a distinction between perceptual- and decision-level suppression because participants made responses to the target so that suppression of the distractor may either reflect the reduced probability of containing the target or reduced sensory evidence. To solve this problem, Gaspelin et al. (2015) used the probe letter task where participants reported the identity of probe letters on all stimuli, not just the target (Gaspelin & Luck, 2018a; Gaspelin et al., 2015; Stilwell & Gaspelin, 2021; Wang & Theeuwes, 2020). The probe letter task was interleaved with regular search trials but was administered less frequently than the search task to avoid changes in participants’ search strategy. On probe trials, letters were briefly presented on all stimuli of the search display and participants were asked to identify as many letters as possible. Performance was best for letters shown on the target shape, as would be expected if participants followed the instruction to look for the target shape. More interestingly, letter identification was worse on the distractor than on nontarget stimuli, consistent with the idea that distractor processing was suppressed below baseline (see also Chang & Egeth, 2019, 2020). Because the probe letters were only briefly presented and masked, the worse performance with distractor than with nontarget stimuli appears to reflect perceptual-level suppression of the distractor. That is, the perceptual quality of letters on the distractor stimulus may have been worse because the distractor was attentionally suppressed.

Recently, however, Lien et al. (2021) provided evidence against perceptual-level suppression of salient stimuli in the probe letter paradigm. Lien et al. (2021) showed that probe performance was worse on any nontarget stimulus that could be excluded from search, regardless of whether it was salient. In particular, Lien et al. (2021) compared displays with a single stimulus in the distractor color to displays in which several stimuli were in the distractor color. If attentional suppression was deployed to counter capture from salient elements, the probe suppression effect should be stronger with a single than with multiple distractors because only the single stimulus was salient. However, letter identification on stimuli in the distractor color was reduced for salient and non-salient distractors, which is inconsistent with perceptual-level suppression of salient distractors. Rather, the reduced performance could be ascribed to strategic behavior and decision-level suppression. In the search task, the target shape was never in the distractor color. Therefore, participants may have suppressed the distractor during decision-making in the probe letter task, which may have decreased letter identification compared to non-targets sharing the target color.

In the current contribution, we explored report bias as another decision-level mechanism resulting in worse performance on distractor than on nontarget stimuli. In this study, we focused on the small search displays shown in Fig. 1. In the probe letter task, participants were free to choose the locations to report and it cannot be ruled out that they reported letters on some locations less frequently. For instance, there may have been a bias against reporting the letter on the distractor location because the target shape could never be in the distractor color. A bias against reporting letters on the distractor would result in worse performance compared to nontargets in the majority color. As laid out in the results section, only about 61%–94% of the reported letters in the probe task were correct. Because letter report was free, it is not possible to match the wrong letters to the actual letters shown on the target, non-target or distractor stimuli. Therefore, it is not possible to evaluate report bias in the original version of the probe letter task. However, changes in the frequency of reports are likely to occur. For instance, an exogenous cue (i.e., a flash in the periphery) may induce a bias to select the cued location in an unrelated spatial decision task (Danziger & Rafal, 2009). This selection bias may result in the spurious impression that perception improved at the location of the cue. For instance, Shiu and Pashler (1994) found improved performance at the cued location, but only when participants were uncertain about where the target had appeared. In this situation, the cue may have biased participants to report the stimulus at the cued location more frequently, which improved performance at this location. In contrast, without uncertainty about the target location, performance at the cued location was comparable to performance at un-cued locations, suggesting that the cue did not enhance perceptual-level processes. Thus, uncertainty about which stimulus to report may result in a bias to report the cued stimulus. To prevent such report bias, studies on exogenous cueing used response cues which instructed participants to report the probe stimulus at a certain location (see p. 1493 in Carrasco, 2011). Response cues eliminate uncertainty about which location to report. Further, response cues keep the working memory load at a single item, which avoids potential biases in the transfer or maintenance of letters in visual working memory.

In the current contribution, we compared three probe tasks that aimed at measuring perceptual performance on the target, distractor and nontarget locations. Experiment 1 replicated the standard probe task from Gaspelin et al. (2015), whereas Experiments 2 and 3 used modified probe tasks with a response cue to remove location uncertainty and reduce report bias. If a report bias against distractor stimuli contributed to distractor suppression, then we expect the difference between distractor and nontarget stimuli to decrease from the original to the modified probe tasks. Our argument is similar to Shiu and Pashler’s (1994) in that we suggest that a report bias may arise from location uncertainty combined with the occurrence of a salient event. Unlike Shiu and Pashler (1994), however, we argue that a salient distractor in feature search is less likely to be reported, whereas their study suggested that that a cued stimulus was more likely to be reported.

## Experiment 1: free recall

Experiment 1 served to replicate the standard findings from the probe letter task. We introduced three changes to make the task more similar to Experiments 2 and 3. First, participants had to indicate at least one letter, which makes the task more similar to the forced choice task in Experiments 2 and 3. In previous studies, participants were allowed to not respond in the probe task, but it is unclear how often this occurred. Second, feedback was given after each trial. In previous studies, no or only summary feedback was given. We provided feedback to motivate participants and allow them to optimize their performance, but we do not know whether this worked as expected. Third, we changed the percentages of distractor-present and -absent trials to 60–40 instead of 50–50. This was necessary to have a sufficient number of probe trials for the distractor in Experiments 2 and 3.

## Methods

### Participants

The study was modeled after Experiment 3 in Gaspelin et al. (2015), which had 24 subjects. We increased the sample size from 24 to 40 in this and the following experiment because it was likely that some datasets would be lost because of ceiling effects. The effect size for the difference between letter identification at the nontarget and distractor locations in Experiment 3 of Gaspelin et al. (2015) was Cohen’s *d*_*z*_ = 1.14. Similarly, Lien et al. (2021) reported a Cohen’s *d*_*z*_ = 0.95. With a sample size of 40 and a Cohen’s *d*_*z*_ of 0.95, G*Power 3 (Faul et al., 2007) indicates a minimal *t*(39) = 1.68 (bilateral) and a power of 0.99. There were 40 undergraduate psychology students in Experiment 1 (9 male; age: *M* = 21.3 years, *SD* = 1.8). Students participated for class credit or were paid 20 Swiss Francs. They reported normal or corrected-to-normal vision.

### Apparatus

To display the stimuli, we used a 22.5-inch VIEWPixx Light (VPixx Technologies Inc., Saint-Bruno, Canada) at a temporal resolution of 100 Hz and a spatial resolution of 1920 × 1200 pixels. Color coordinates and luminance were measured by a ColorCAL MKII colorimeter (Cambridge Research Systems, Rochester, UK). Participant’s head was restrained by a chin/forehead rest at a distance of 66 cm. The experiment was run by the Psychtoolbox (Kleiner et al., 2007). Responses were collected with a mouse.

### Stimuli

A black fixation cross (0.5° × 0.5°, linewidth 0.07°) was shown unless otherwise noted. One geometric shape was shown left, right, above and below the fixation cross at a distance of 2° (center-to-center). The circle had a diameter of 1.4°. The square and diamond had a side length of 1.2°. The hexagon had horizontal and vertical diameters of 1.3° and 1.5°, respectively. The dots were squares with a side length of 0.1° and were shown 0.5° to the left or right of the center of the geometric shapes. As probe stimuli, letters in Arial (height of 0.7°) were shown. To avoid that any of the letters would stand out in the probe display because of its size, we applied the following selection criteria. The letters were selected to have a width between 0.5° and 0.7° and a total number of lit pixels between 1280 and 1680. In addition, the letter Q was removed because of its similarity to the letter O. The 21 letters fulfilling these criteria were A, B, C, D, E, F, G, H, K, L, N, O, P, R, S, T, U, V, X, Y, Z. The hash sign was used as mask. The response display showed a 5 × 5 matrix with the 21 possible letters in alphabetical order and four fillers (%, ?, &, $) at the end to have a square matrix. The spacing between letters in the response display was 1.3° (center-to-center). Feedback in the probe task was provided by surrounding the correct letters with a circle (diameter of 1.3°) for 1000 ms. Feedback about wrong responses in the search task was provided by a short flicker of the fixation mark (50 ms on/off in three cycles).

The geometric shapes were shown in one of two colors selected in CIELAB-space on an isoluminant color wheel (luminance of 58.8 cd/m^2^ or L* = 61, saturation of 59, rotation of 0° and 180°). The xyY coordinates were red (0.390, 0.303, 57.8) and green (0.179, 0.408, 57.8) with Y in cd/m^2^. The stimuli were presented against a medium gray background (0.282, 0.357, 28.7). The dots and letters were shown in the same gray as the background. The letters in the response display were shown in light gray (0.28, 0.355, 61.7). Selected letters and mouse-overs in the response display were indicated in bright red (0.639, 0.313, 58.6).

### Design

The search task was performed on 70% of the trials and the letter probe task on 30%. To increase the number of task switches that would promote effects of search on probe identification, we limited the number of repetitions of the search task to seven (for a discussion of this issue, see Supplementary Material). The distractor was present on 60% of the trials and absent on 40%. On distractor-absent trials, all shapes were shown in the same color. On distractor-present trials, a randomly determined nontarget shape was shown in a different color. The locations of target and non-target shapes were random. Similarly, the location of the dot inside each shape was random with the constraint that there be two dots on the left and two on the right. The letters in the probe task were selected randomly without replacement from the 21 available letters. The target shape (circle or square) and the majority color (red or green) were fixed for each participant and counterbalanced across participants. After 100 trials, feedback about the percentage of correct responses in each task was displayed for at least 2 s in a self-terminated break. Participants worked through three blocks of 200 trials. Thus, there were 108 probe + 252 search trials = 360 trials with distractor and 72 probe + 168 search trials = 240 trials without distractor.

### Procedure

A trial started with the presentation of the fixation cross for randomly 700–1050 ms. On search trials, the search display appeared and remained on the screen until a response was collected. Participants were instructed to locate the target shape and indicate the dot location by mouse click. Left and right dot locations were mapped onto left and right mouse buttons, respectively. Participants were told to respond as rapidly and accurately as possible while making less than 10% errors. They were informed that the target shape would never be shown in the singleton color. On probe trials, the search display was shown for 200 ms before the probe letters were superposed for 100 ms and then masked for 200 ms. Participants were instructed to identify the probe letters in the following response display. To select and unselect letters in the response display, they pressed the left mouse button. To indicate that they were done, they pressed the right mouse button. Participants were forced to choose at least one letter and were encouraged to guess if they were unsure. They were also told that speed did not matter in the probe task.

Before the experiment started, participants performed three training blocks. In the first and second training blocks, the search and probe tasks were performed separately. In the third training block, the tasks were mixed as in the experiment. Participants practiced the task until they felt comfortable with it, but at least for 50 trials in training blocks 1 and 3, and for 25 trials in training block 2.

## Results

**Fig. 2:**
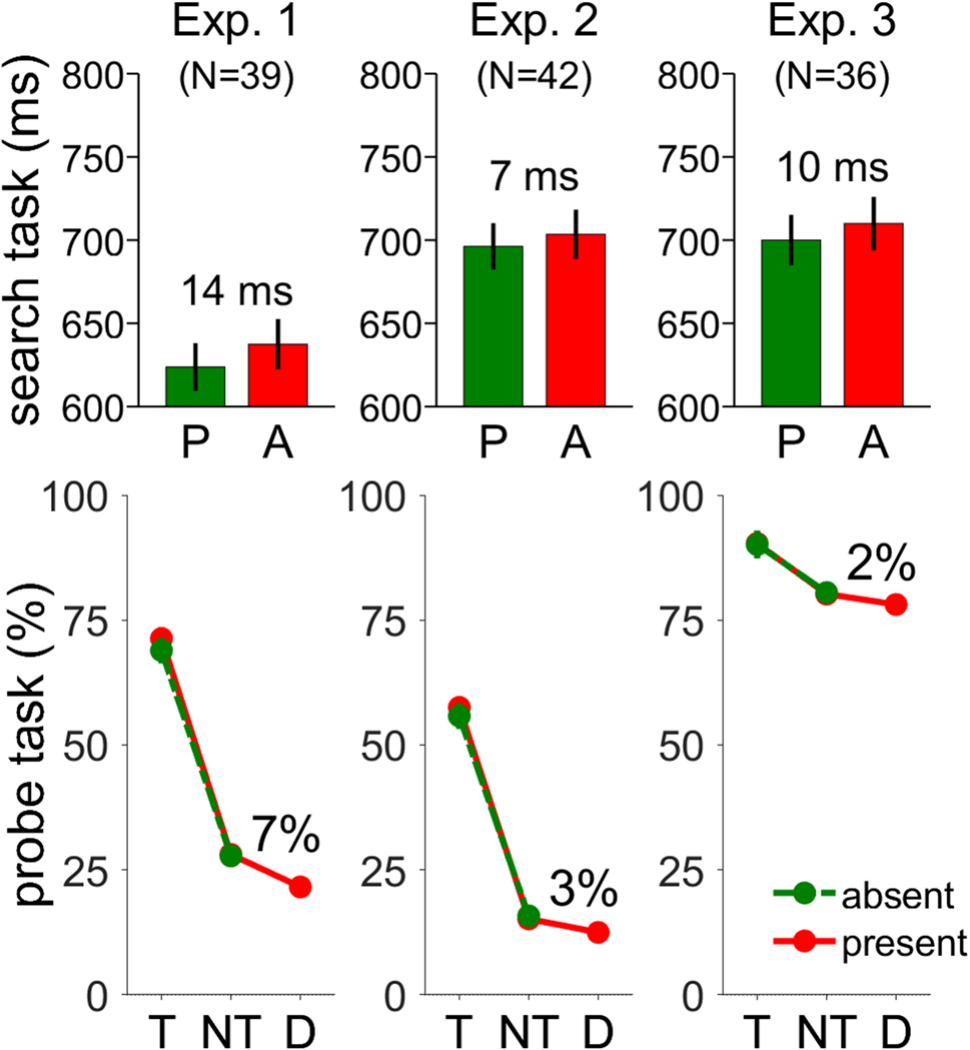
Performance in the search and probe tasks in Experiments 1–3. The upper panels show search reaction times (RT) on distractor-absent and distractor-present trials. The lower panels show the percentage of correct letters in the probe task. Performance was probed on the target position, the nontarget positions, and the distractor position (if present). Error bars show the between-participant standard error of the mean, which was smaller than the symbols in the lower panels

**Fig. 3:**
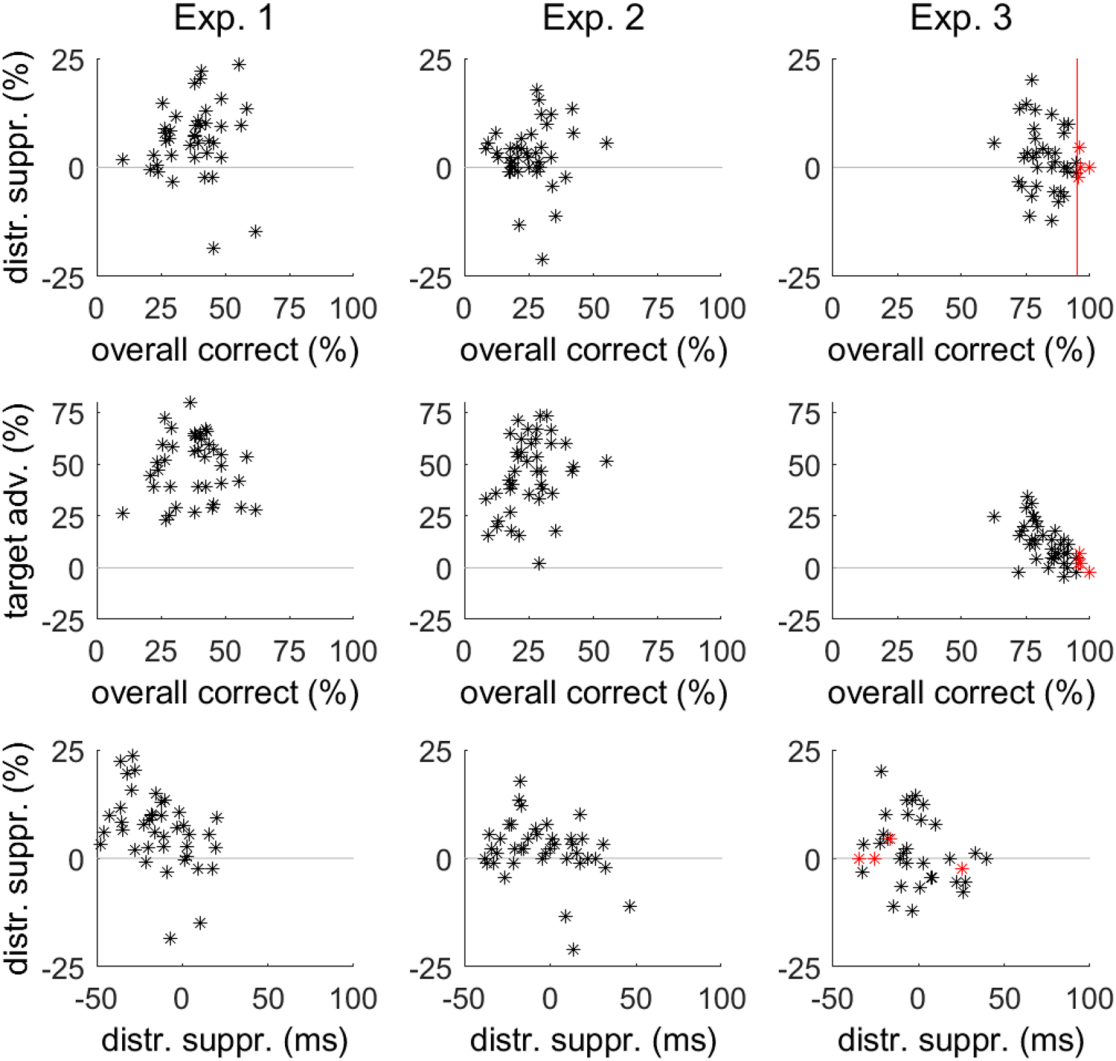
Scatter plots of individual means in Experiments 1–3. Overall correct probe identification was the mean percentage of correct responses across all stimuli in distractor-present and -absent trials. The suppression effect was calculated separately for probe and search tasks. For the probe task, distractor suppression was the difference between nontarget and distractor stimuli in percentage correct (%). For search reaction times, distractor suppression was the difference in reaction times between distractor-present and -absent trials in milliseconds (ms). In addition, we calculated the target advantage as the difference between target and nontarget stimuli in the probe task. For Experiment 3, datasets not included in the analyses are indicated in red and the limit for inclusion is shown by the red vertical line

For the search task, we removed trials with RTs longer than 2000 ms and trials with choice errors. This resulted in the loss of 0.3% and 2% of trials, respectively. Mean RTs and probe performance are shown in Fig. 2 and scatterplots are shown in Fig. 3. Consistent with distractor suppression, RTs in the search task were 14 ms shorter on distractor-present than -absent trials (624 vs. 637 ms), *t*(39) = 4.68, *p* < 0.01, Cohen’s *d*_*z*_ = 0.74.

In the probe task, participants selected 2.5 letters on average and 61% of these letters were correct. In comparison, 1.7 letters were selected with 94% correct in Experiment 3 of Gaspelin et al. (2015), 2.1 letters with 63% correct in Experiment 1 of Gaspelin and Luck (2018a), and 1.6 letters with 71% correct in Experiment 1 of Lien et al. (2021). For convenience, the number of selected letters and percentage of correct letters can be converted into the number of correct letters. In the current study, 1.5 letters were correctly recalled compared to 1.6 letters in Gaspelin et al. (2015), 1.3 in Gaspelin and Luck (2018a), and 1.1 in Lien et al. (2021). Thus, performance in the current study was comparable to previous studies.

To assess distractor suppression in the probe task, we compared percentages of correct identifications for letters shown on distractor and non-targets shapes. Only distractor-present trials were considered. We found lower percentages for letters on the distractor than on nontarget shapes (21.6% vs. 28.1%), *t*(39) = 4.86, *p* < 0.01, Cohen’s *d*_*z*_ = 0.77, yielding a mean suppression score of 6.5%. Our suppression score appears smaller than the suppression score of 12% reported by Gaspelin et al. (2015), but comparable to the 7% reported by Gaspelin and Luck (2018a) and the 8% reported by Lien et al. (2021)

Next, we evaluated differences between probe letter identification at target and nontarget locations as a function of distractor presence. We subjected individual percentage of correct responses to a 2 (distractor: present, absent) × 2 (shape: target, nontarget) ANOVA. The percentage of correct identifications was 1.4% larger on distractor-present than -absent trials (49.7% vs. 48.3%), *F*(1, 38) = 8.20 *p* < 0.01, η_p_^2^ = 0.174, confirming that suppression of the distractor freed resources for the processing of target and nontarget stimuli as suggested by Gaspelin et al. (2015). In addition, performance was 42.1% better for letters on the target shape than for letters on nontarget shapes (70.1% vs. 27.9%), *F*(1, 39) = 190.95, *p* < 0.01, η_p_^2^ = 0.882, suggesting that the probe task reflected the expected allocation of attention to the target shape. We refer to the difference between target and nontarget shapes as target advantage score. The interaction was not significant, *F*(1, 39) = 2.22, *p* = 0.145, η_p_^2^ = 0.054.

## Discussion

The present experiment replicated the main results from Gaspelin et al. (2015) with our adjustments of the probe task. First, RTs in the search task were shorter on distractor-present than -absent trials. Second, letter identification in the probe task was worse for letters on the distractor compared to nontarget stimuli suggesting that the processing of distractor stimuli was suppressed below baseline. Third, identification performance was better for letters on the target compared to letters on nontargets. The size of the effects was comparable to previous studies, showing that the addition of trial-by-trial feedback, the restricted run length, the smaller set of probe letters, the requirement to indicate at least one letter and the larger percentage of distractor-present trials did not alter the results. Concerning the latter point, it is possible that only more extreme variations of distractor prevalence result in changes of the distractor effect. For instance, it was demonstrated that RTs on distractor-present trials increased more strongly with 20% distractor-present trials compared to 50% or 80% (Geyer et al., 2008; Müller et al., 2009). However, it is difficult to draw firm conclusions from prior research because we observed shorter RTs on distractor-present compared to distractor-absent trials, whereas previous research found the opposite.

## Experiment 2: response cue with masked letters

In Experiment 2, we changed the probe letter task to reduce report bias and memory load (see Fig. 1). A response cue appeared directly after presentation of the probe stimulus and participants were asked to report the letter in the direction of the cue.

## Methods

Forty-four undergraduate psychology students participated, but only 42 participants were retained in the final sample (4 male; age: *M* = 21.5 years, *SD* = 3.4). The methods were the same as in Experiment 1 except that the probe task was different. On probe trials, the probe display was followed by the response cue, which remained on the screen until a response was registered. As a response cue, the fixation cross extended one of its branches by 0.2° toward one of the stimuli and participants had to report the letter at the indicated location. Participants selected a single letter in the response display and confirmed their choice by a left mouse click. In the response display, the response cue was superposed on the letter “P”, but remained visible because it was black whereas the letters were gray. The same design as in Experiment 1 was used except that participants performed 1000 instead of 600 trials. More trials were necessary because target, distractor and nontarget stimuli were probed on different trials, and not simultaneously as with free recall. There were 300 probe trials which were distributed as follows. On distractor-present trials, there were 45 trials for the target shape, 45 trials for the distractor shape and 90 trials for the two nontarget shapes. On distractor-absent trials, there were 30 trials for the target and 90 trials for the nontarget shapes.

## Results

Two participants did not follow the instructions to keep the error rate in the search task below 10%. Therefore, their datasets were discarded. For the remaining data from the search task, we removed trials with RTs longer than 2,000 ms and trials with choice errors. This resulted in the loss of 1.0% and 1.9% of trials, respectively. Consistent with distractor suppression, RTs in the search task were shorter by 7 ms on distractor-present than -absent trials (696 vs. 703 ms), *t*(41) = 1.93, *p* = 0.06, Cohen’s *d*_*z*_ = 0.30. The group means are shown in Fig. 2 and a scatterplot with individual means is shown in Fig. 3 (bottom row). To evaluate differences between Experiments 1 and 2, we conducted a 2 (Experiment: 1, 2) × 2 (distractor: present, absent) mixed ANOVA on RTs. We confirmed the shorter RTs in the presence than in the absence of a distractor (660 vs. 670 ms), *F*(1, 80) = 19.00, *p* < 0.01, η_p_^2^ = 0.192. Further, RTs were shorter in Experiment 1 than 2 (631 vs. 700 ms), *F*(1, 80) = 12.76, *p* < 0.01, η_p_^2^ = 0.138. Possibly, participants delayed their responses in the search task to make sure that the response cue for the probe task was not shown. Importantly, the effect of distractor presence was not modulated by experiment, *F*(1, 80) = 1.77, *p* = 0.19, η_p_^2^ = 0.022, showing that the delay induced by the response cue in Experiment 2 did not change distractor processing.

To assess distractor suppression in the probe task, we compared percentages of correct identifications for letters shown on distractor and non-targets shapes. Only distractor-present trials were considered. The difference of 2.7% between distractor and non-targets was significant (15.2% vs. 12.5%), *t*(41) = 2.44, *p* = 0.02, Cohen’s *d*_*z*_ = 0.38. However, suppression scores were smaller than with free recall in Experiment 1 (2.7% vs. 6.5%), *t*(80) = 2.23, *p* = 0.03, Cohen’s *d*_*s*_ = 0.49. Because overall performance on the probe task was lower in Experiment 2 than 1, it may be that the reduced suppression scores resulted from floor effects. Therefore, we excluded four participants with mean performance below 5% in the critical distractor and nontarget conditions. However, the results for the remaining participants were unchanged, which argues against floor effects.

Next, we evaluated differences between probe letter identification at target and non-target locations as a function of distractor presence. We subjected individual percentage of correct responses to a 2 (distractor: present, absent) × 2 (shape: target, nontarget) ANOVA. The percentage of correct identifications was similar on distractor-present and -absent trials (36.3% vs. 35.8%), *F*(1, 32) = 0.25, *p* = 0.62, η_p_^2^ = 0.006, which is at odds with the results from Experiment 1 and Gaspelin et al. (2015). Further, performance was better by 41.2% for letters on the target shape than for letters on the nontarget shapes (56.7% vs. 15.5%), *F*(1, 32) = 219.61, *p* < 0.01, η_p_^2^ = 0.843, showing that the probe task was sensitive to the expected deployment of visual attention. The interaction was not significant, *F*(1, 32) = 1.86, *p* = 0.18, η_p_^2^ = 0.043.

Further, we compared the target-advantage scores from Experiments 1 and 2 by independent samples t-test. Target-advantage scores were similar in Experiments 1 and 2 (42.2% vs. 41.2%), *t*(80) = 0.79, *p* = 0.79, Cohen’s *d*_*s*_ = 0.06. The overall percentage of correct letters was higher in Experiment 1 than 2 (37.6% vs. 25.4%), *t*(80) = 5.36, *p* < 0.01, Cohen’s *d*_*s*_ = 1.18.

## Discussion

We used the same stimuli and search task as in Experiment 1 but modified the probe task. Instead of the free recall procedure introduced by Gaspelin et al. (2015), we presented a response cue after the probe display and participants had to report the letter indicated by the cue. Thereby, we removed uncertainty about which stimulus to report and prevented differences in the frequency of reports (i.e., report bias). Experiment 2 replicated two key findings from Experiment 1. First, search RTs were shorter on distractor-present than -absent trials, consistent with the standard distractor-suppression effect. While this effect was not quite significant with two-sided testing (*p* = 0.06) in Experiment 2, it would be justified to perform a one-sided test given the strong expectations derived from Experiment 1 and the literature. In this case, statistical significance would be reached. Second, performance was better for probes on the target compared to probes on nontarget stimuli, showing that the probe task reflected the expected attentional selection of the search target. These findings confirm that the modified probe task did not change basic characteristics of the tasks. However, the modified probe task deviated in an important aspect from the original probe letter task. Notably, the difference between distractor and nontarget stimuli was strongly reduced. Because the improved performance at the target location was unchanged, whereas the worse performance at the distractor location was reduced, the two effects may have different origins. The improved performance at the target location is likely to reflect perceptual enhancement (Carrasco, 2011) or noise exclusion (Dosher & Lu, 2000), whereas the worse performance at the distractor location may partially reflect report bias or other forms of decision-level suppression. Consistently, Experiment 2 showed a reduction of probe suppression scores, but the difference between nontarget and distractor stimuli was not completely eliminated. It could be that the remaining difference reflects perceptual-level suppression, but other explanations may apply (see General Discussion). One issue with the current procedure is that there were many changes in the periphery occurring around the onset of the response cue, which may have delayed its perception. Delayed perception of the response cue may have reduced performance on the probe task because participants failed to transfer the probed letter into visual working memory before it was erased from iconic memory (Sperling, 1960). To remedy this shortcoming, we removed the peripheral onsets around the time of the response cue and simplified the stimulus displays.

## Experiment 3: response cue with binary choice

The task was modeled after previous research on attentional enhancement of perception by endogenous and exogenous cues (e.g., Dosher & Lu, 2000; Ling & Carrasco, 2006a, 2006b). To this end, only two possible probe letters were used (“o” and “ + ”). For ease of exposition, we refer to the two shapes as letters even though “ + ” is a symbol. Unlike in Experiments 1 and 2, performance was limited by the short presentation time (similar to Ling & Carrasco, 2006a). In pilot testing, we found that performance dropped to chance level with efficient masks (e.g., “ + ” and “o” superposed), which is why we did not include a mask. Note that the hashtag masks in Experiments 1 and 2 were inefficient as there was little correspondence between the features of the letters and the mask. Masking increases with featural similarity between target and masks (e.g., Hermens & Herzog, 2007).

## Methods

Forty students participated and 36 datasets were retained (4 male; age: *M* = 22.6 years, *SD* = 5.7). The methods were the same as in Experiment 2 except that the probe task was different. On probe trials, the duration of the probe display was reduced from 100 to 70 ms to avoid ceiling effects. The probe display was followed by the response cue, which remained on the screen until a response was registered. The letters in the probe display were “o” and “ + ” with diameters of 0.7°, drawn in a pen width of 0.07°. Two letters of each kind were randomly assigned to the stimuli. Responses were collected on a RESPONSEPixx Handheld 5-button response box (VPixx Technologies Inc., Saint-Bruno, Canada). Participants pressed the left button for “o” and right button for “ + ”. The same buttons were also used for the search task where participants pressed the left button for a dot on the left and the right button for a dot on the right.

## Results

The percentage of correct probe responses was close to ceiling for some participants. As ceiling effects reduce variability (see red data points in the right column of Fig. 3), we removed four datasets from analysis. As a criterion for exclusion, we chose 95% of overall correct responses. Keeping these subjects in the analysis or arcsine-transforming individual means did not change the results.

For data from the search task, we removed trials with RTs longer than 2,000 ms and trials with choice errors. This resulted in the loss of 0.7% and 1.8% of trials, respectively. Consistent with distractor suppression, RTs in the search task were shorter by 10 ms on distractor-present than -absent trials (700 vs. 710 ms), *t*(35) = 2.19, *p* = 0.02, Cohen’s *d*_*z*_ = 0.37. The group means are shown in Fig. 2 and a scatterplot with individual means is shown in Fig. 3 (bottom row). To evaluate differences between Experiments 1 and 3, we conducted a 2 (Experiment: 1, 3) × 2 (distractor: present, absent) mixed ANOVA on RTs. We confirmed the shorter RTs in the presence than in the absence of a distractor (662 vs. 674 ms), *F*(1, 74) = 19.98, *p* < 0.01, η_p_^2^ = 0.213. Further, RTs were shorter in Experiment 1 than 3 (631 vs. 705 ms), *F*(1, 74) = 13.72, *p* < 0.01, η_p_^2^ = 0.156. As in Experiment 2, participants may have waited for the response cue to confirm its absence before responding to the search target. Importantly, the effect of distractor presence was not modulated by experiment, *F*(1, 74) = 0.52, *p* = 0.47, η_p_^2^ = 0.007, showing that the deployment of attention was not affected by the response cue.

To assess distractor suppression in the probe task, we compared percentages of correct identifications for letters shown on distractor and non-targets shapes. Only distractor-present trials were considered. The difference of 2.1% between distractor and non-targets was not significant (78.1% vs. 80.0%), *t*(35) = 1.7, *p* = 0.10, Cohen’s *d*_*z*_ = 0.28. Because visual inspection of Fig. 3 (top row) suggests that the variability in individual distractor suppression scores was similar between Experiments 1 and 3, we compared suppression scores from Experiment 1 and 3 by independent samples t-test. Suppression scores were smaller with the response cue in Experiment 3 than with free recall in Experiment 1 (2.1% vs. 6.5%), *t*(74) = 2.37, *p* = 0.02, Cohen’s *d*_*s*_ = 0.55. Note that the variance of the suppression scores was similar according to Levene’s test, *F* < 0.1, *p* = 0.85.

Next, we evaluated differences between probe letter identification at target and nontarget locations as a function of distractor presence. We subjected individual percentage of correct responses to a 2 (distractor: present, absent) × 2 (shape: target, nontarget) ANOVA. The percentage of correct identifications was similar on distractor-present and -absent trials (85.3% vs. 85.3%), *F*(1, 32) < 0.01, *p* = 0.94, η_p_^2^ < 0.001, which is at odds with the results from Experiment 1 and Gaspelin et al. (2015). Further, performance was better by 9.9% for letters on the target shape than for letters on the nontarget shapes (90.2% vs. 80.3%), *F*(1, 32) = 58.88, *p* < 0.01, η_p_^2^ = 0.627, showing that the probe task was sensitive to the expected deployment of visual attention. The interaction was not significant, *F*(1, 32) = 0.12, *p* = 0.736, η_p_^2^ = 0.003.

Further, we compared the target-advantage scores from Experiments 1 and 3 by independent samples t-test. Target-advantage scores were larger in Experiment 1 than 3 (42.2% vs. 9.9%), *t*(58.2) = 11.60, *p* < 0.01, Cohen’s *d*_*s*_ = 2.86. Levene’s test showed that the variance of these scores was larger in Experiment 1 than 3 (15.6 vs. 7.7), *F* = 18.79, *p* < 0.01, which can be appreciated in the scatter plots of Fig. 3 (center row). Thus, the smaller target-advantage scores in the current experiment may reflect the reduced range of overall performance. In Experiment 1, performance could vary between 0 and 100%, whereas it is expected to vary only between 50 and 100% in Experiment 3. The difference in scale was reflected in the size of the target advantage and the variability of this effect. In contrast, the variability of the suppression scores was the same in Experiments 1 and 3.

Finally, we performed additional analyses to evaluate whether the difference between distractor and nontarget shapes was modulated by the correspondence of the probe letters, task switching, or repetition of the distractor. These results are reported in the Supplementary Material and do not affect the conclusion drawn from the results presented in the main text.

## Discussion

We modified the displays to avoid peripheral stimulation around the presentation of the response cue. As a result, it may have been easier for participants to focus on the response cue. The results were similar to Experiment 2. We replicated shorter search RTs on distractor-present than -absent trials and probe performance was better on the target than on nontarget stimuli. These findings confirm that the new probe task in Experiment 3 did not change basic characteristics of the search tasks. However, Experiment 3 confirmed that the worse performance on the distractor location in the probe task was strongly reduced when report bias was avoided. Notably, probe performance was no longer reliably different between target and nontarget stimuli. A possible objection to this conclusion is that the expected range of percentage correct values was reduced in the modified compared to the original probe task. As a result, any differences between stimuli would be smaller. However, the smaller differences are expected to be accompanied by smaller variances. This was true for the target-advantage scores, but not for the suppression scores. For the suppression scores, the variance was similar in the modified and original versions of the task. Possibly, the modified version of the task was more sensitive to individual differences in attentional deployment, which compensated for the smaller range. Regardless of range, the effect sizes show that the suppression effect was strongly reduced from Cohen’s *d*_*z*_ = 0.77 in Experiment 1 to Cohen’s *d*_*z*_ = 0.28 in Experiment 3. These results suggest that decision-level suppression (i.e., report bias) contributed to the worse performance for distractor compared to nontarget stimuli in the original probe letter task. In contrast, effects of perceptual-level suppression are not expected to change when report bias is avoided. Possibly, salient distractors had only little effect on early perceptual-level processing, consistent with the ability to ignore distractors occurring at irrelevant locations (Ruthruff & Gaspelin, 2018; but see Burnham, 2018).

## General Discussion

We re-examined results from small search displays that provided evidence for the suppression of salient-but-irrelevant distractor stimuli. In the current contribution, we tried to disentangle perceptual- from decision-level suppression. We argued that perceptual-level suppression should be impervious to report bias, whereas decision-level suppression should be susceptible. We argued that the free report in the original probe letter task allowed for report bias, which is a decision-level process, and therefore conducted two experiments with a response cue to reduce report bias. Replicating previous research, we found that search performance was better in the presence than in the absence of a color distractor (Chang & Egeth, 2019, 2020; Cunningham & Egeth, 2016; Gaspelin & Luck, 2018a; Gaspelin et al., 2015; Kerzel & Burra, 2020; Lamy et al., 2006; Moher & Egeth, 2012). However, our main interest was in the probe letter task which required participants to recall as many letters as possible from an array of letters presented briefly on the search display. Identification performance was found to be better for letters on the target than on nontarget stimuli, which reflects the top-down task set and has also been confirmed in the contingent capture paradigm (Burnham, 2020). The critical point was performance for letters on the distractor stimulus. According to the idea of suppression, identification of letters on the distractor should be worse than identification of letters on nontarget stimuli. Identification of letters on nontarget stimuli is assumed to represent the baseline level of processing. We observed that the difference between distractor and nontarget stimuli depended on the way to report the letters, which confirms that decision-level factors such as report bias were involved.

Our results show that suppression scores for the distractor were larger in the original version of the probe task than in our modified versions. In the modified versions, a response cue indicated which stimulus had to be reported. In contrast, participants were free to recall any stimulus in the original version, which allowed for biases in decision-level processes. For instance, participants may have reported the stimulus at the distractor location less frequently because the distractor was a color that was irrelevant in the more frequent search task. As a result, performance for probes at the distractor location may have been worse than performance at the nontarget location. At the same time, the results from Experiment 2 confirm that the improved performance on the target location was not affected by changes in the probe procedure. In Experiment 2, the improvement of performance at the target location with a response cue was similar to the improvement with the free recall procedure in Experiment 1. Thus, it is likely that the improved performance at the target location reflects perceptual-level enhancement, which resists changes in the probe procedure, whereas the worse performance on the distractor location reflects decision-level report bias to some degree, which is susceptible to changes in the probe procedure.

While the worse performance at the distractor compared to the nontarget locations was greatly reduced in Experiments 2 and 3 compared to Experiment 1, it was statistically significant in Experiment 2 and approached significance in Experiment 3, with no difference between the latter experiments, *t*(76) = 0.33, *p* = 0.75. The question is whether perceptual- or decision-level suppression accounts for the remaining difference. While we can be sure that response cues reduced report bias, it is unlikely that response cues excluded all decision-related processes. For instance, attention was distributed across the peripheral locations at the start of a probe trial, but the response cue appeared in the center, which required a refocusing of attention on central fixation. It may be safe to assume that the refocusing of attention and the interpretation of the response cue took time. After this delay, it was necessary to retrieve the information about the cued stimulus from short-term memory. Given that the distractor never contained useful information in the search task, transfer of information from the distractor location to short-term memory or the retrieval of this information may have been suppressed. Possibly, these biases may be avoided by presenting the response cue at the same time as the probe (e.g., Dosher & Lu, 2000; Ling & Carrasco, 2006b). Besides decision-level mechanisms, however, it may also be possible that the residual difference between distractor and non-targets was indeed caused by perceptual-level suppression. More research is required to decide between these alternatives.

Together with the work of Lien et al. (2021), the present contribution casts doubt on the assumption that salient-but-irrelevant stimuli are suppressed at a perceptual level to avoid attentional capture (see also Kerzel et al., 2021). Similarly, recent studies have questioned electrophysiological evidence for attentional suppression. In electrophysiology, the P_D_ component is a contralateral positivity occurring between 100 and 400 ms after stimulus onset at posterior electrodes PO7/PO8 (Burra & Kerzel, 2013; Gaspar & McDonald, 2014; Hickey et al., 2009; Liesefeld et al., 2021). The P_D_ was associated with distractor suppression and has also been measured in response to the small search displays shown in Fig. 1. Gaspelin and Luck (2018a) reported a P_D_ to the distractor, which may be taken as evidence for distractor suppression (see also Feldmann-Wustefeld et al., 2020). However, a recent investigation found that the P_D_ component to the distractor stimulus was followed by an N2pc component (Kerzel & Burra, 2020). Because the N2pc component is considered a marker of attentional selectivity (Eimer, 1996; Luck & Hillyard, 1994), one would have to conclude that attentional suppression was followed by attentional capture, which is at odds with the attentional suppression hypothesis stating that attentional capture is prevented. While the exact reasons for the sequence of P_D_ and N2pc are debated (Drisdelle & Eimer, 2021; Gaspelin et al., 2022; Kerzel & Burra, 2020), there are doubts that the P_D_ will provide unequivocal evidence for attentional suppression (see also Forschack et al., 2022). The reason is that the P_D_ is the mirror-image of the N2pc component as it occurs in about the same time interval at the same electrodes. That is, a P_D_ to a distractor on one side of the screen could also be an N2pc to a stimulus on the opposite side.

In sum, we show that decision-level mechanisms such as report bias contribute to results from the probe letter task. While we cannot rule out perceptual-level suppression, a look at the literature shows that only a small number of classical approaches include distractor inhibition as a necessary component (Treisman & Sato, 1990; reviewed by Dent et al., 2012). Rather, classic theories give more importance to mechanisms that guide attention toward target stimuli. For instance, biased competition theory (Desimone & Duncan, 1995) assumes that stimuli compete for selection and that both stimulus-driven saliency and the match to a target template in working memory bias this competition. Similarly, the locations (Wolfe, 2021) or weights (Bundesen, 1990; Schneider, 2013) of stimuli or features are increased if they match the target template stored in working memory. Therefore, attentional suppression may not be necessary to enable successful selection.

## Supplementary Information

Below is the link to the electronic supplementary material.

Supplementary file1 (PDF 207 KB)

## Declarations

### Open Practices Statement

Neither of the experiments reported in this article was formally preregistered. The data are available at https://osf.io/c8t39/ and requests for the program code can be sent via email to DK.

### Public significance statement

Human observers are bombarded by visual stimuli. It is therefore vital to select only the relevant stimuli for further processing and to prevent salient-but-irrelevant stimuli from being erroneously selected. Previous research suggested that human observers may suppress these salient-but-irrelevant stimuli. We clarify that distracting stimuli were suppressed in decision-related processes, but effects of perceptual-level processes cannot be ruled out.

## References

[CR1] Adams OJ, Ruthruff E, Gaspelin N (2022). Oculomotor suppression of abrupt onsets versus color singletons. Attention, Perception, & Psychophysics..

[CR2] Awh E, Belopolsky AV, Theeuwes J (2012). Top-down versus bottom-up attentional control: A failed theoretical dichotomy. Trends in Cognitive Sciences.

[CR3] Bacon WF, Egeth HE (1994). Overriding stimulus-driven attentional capture. Perception & Psychophysics.

[CR4] Bundesen C (1990). A theory of visual attention. Psychological Review.

[CR5] Burnham BR (2018). Selectively ignoring locations does not modulate contingent involuntary orienting, but selectively attending does. Visual Cognition.

[CR6] Burnham BR (2020). Evidence for early top-down modulation of attention to salient visual cues through probe detection [journal article]. Attention, Perception, & Psychophysics.

[CR7] Burra N, Kerzel D (2013). Attentional capture during visual search is attenuated by target predictability: Evidence from the N2pc, Pd, and topographic segmentation. Psychophysiology.

[CR8] Carrasco M (2011). Visual attention: The past 25 years. Vision Research.

[CR9] Chang S, Egeth HE (2019). Enhancement and Suppression Flexibly Guide Attention. Psychological Science.

[CR10] Chang S, Egeth HE (2020). Can salient stimuli really be suppressed?. Attention, Perception, & Psychophysics..

[CR11] Cunningham CA, Egeth HE (2016). Taming the white bear: initial costs and eventual benefits of distractor inhibition. Psychological Science.

[CR12] Danziger S, Rafal R (2009). The effect of visual signals on spatial decision making. Cognition.

[CR13] Dent K, Allen HA, Braithwaite JJ, Humphreys GW (2012). Parallel distractor rejection as a binding mechanism in search. Frontiers in Psychology.

[CR14] Desimone R, Duncan J (1995). Neural mechanisms of selective visual attention. Annual Review of Neuroscience.

[CR15] Dosher BA, Lu ZL (2000). Noise exclusion in spatial attention. Psychological Science.

[CR16] Drisdelle BL, Eimer M (2021). PD components and distractor inhibition in visual search: New evidence for the signal suppression hypothesis. Psychophysiology.

[CR17] Eimer M (1996). The N2pc component as an indicator of attentional selectivity. Electroencephalography and Clinical Neurophysiology.

[CR18] Eimer M (2014). The neural basis of attentional control in visual search. Trends in Cognitive Sciences.

[CR19] Faul F, Erdfelder E, Lang AG, Buchner A (2007). G*Power 3: A flexible statistical power analysis program for the social, behavioral, and biomedical sciences. Behavior Research Methods.

[CR20] Feldmann-Wustefeld T, Busch NA, Schubo A (2020). Failed suppression of salient stimuli precedes behavioral errors. Journal of Cognitive Neuroscience.

[CR21] Forschack N, Gundlach C, Hillyard S, Müller MM (2022). Electrophysiological evidence for target facilitation without distractor suppression in two-stimulus search displays. Cerebral Cortex.

[CR22] Gaspar JM, McDonald JJ (2014). Suppression of salient objects prevents distraction in visual search. Journal of Neuroscience.

[CR23] Gaspelin, N., Egeth, H., & Stilwell, B. T. (2022). Electrophysiological Evidence for the Suppression of Highly Salient Distractors. *Journal of Cognitive Neuroscience*. 10.1162/jocn_a_01827 35104346

[CR24] Gaspelin N, Leonard CJ, Luck SJ (2015). Direct evidence for active suppression of salient-but-irrelevant sensory inputs. Psychological Science.

[CR25] Gaspelin N, Leonard CJ, Luck SJ (2017). Suppression of overt attentional capture by salient-but-irrelevant color singletons. Attention, Perception, & Psychophysics.

[CR26] Gaspelin N, Luck SJ (2018). Combined electrophysiological and behavioral evidence for the suppression of salient distractors. Journal of Cognitive Neuroscience.

[CR27] Gaspelin N, Luck SJ (2018). The role of inhibition in avoiding distraction by salient stimuli. Trends in Cognitive Sciences.

[CR28] Geyer T, Müller HJ, Krummenacher J (2008). Expectancies modulate attentional capture by salient color singletons. Vision Research.

[CR29] Hermens, F., & Herzog, M. H. (2007). The effects of the global structure of the mask in visual backward masking. *Vision Research*, *47*(13), 1790–1797. 10.1016/j.visres.2007.02.020 10.1016/j.visres.2007.02.020 17466356

[CR30] Hickey C, Di Lollo V, McDonald JJ (2009). Electrophysiological indices of target and distractor processing in visual search. Journal of Cognitive Neuroscience.

[CR31] Kerzel D, Burra N (2020). Capture by context elements, not attentional suppression of distractors, explains the P_D_ with small search displays. Journal of Cognitive Neuroscience.

[CR32] Kerzel D, Huynh Cong S, Burra N (2021). Do we need attentional suppression?. Visual Cognition.

[CR33] Kleiner M, Brainard DH, Pelli D (2007). What’s new in Psychtoolbox-3?. Perception.

[CR34] Kruger A, Tunnermann J, Scharlau I (2017). Measuring and modeling salience with the theory of visual attention. Attention, Perception, & Psychophysics.

[CR35] Lamy D, Bar-Anan Y, Egeth HE, Carmel T (2006). Effects of top-down guidance and singleton priming on visual search. Psychonomic Bulletin & Review.

[CR36] Lamy, D., Leber, A. B., & Egeth, H. E. (2012). Selective attention. In A. F. Healy & R. W. Proctor (Eds.), *Comprehensive handbook of psychology* (Vol. 4, pp. 265–294). Wiley.

[CR37] Lien MC, Ruthruff E, Hauck C (2021). On preventing attention capture: Is singleton suppression actually singleton suppression?. Psychological Research Psychologische Forschung.

[CR38] Liesefeld, H. R., Liesefeld, A. M., & Muller, H. J. (2021). Preparatory control against distraction is not feature-based. *Cerebral Cortex*. 10.1093/cercor/bhab341 10.1093/cercor/bhab341 34585718

[CR39] Ling, S., & Carrasco, M. (2006a). Sustained and transient covert attention enhance the signal via different contrast response functions. *Vision Research*, *46*(8–9), 1210–1220. 10.1016/j.visres.2005.05.008 10.1016/j.visres.2005.05.008 PMC1557421 16005931

[CR40] Ling S, Carrasco M (2006). When sustained attention impairs perception. Nature Neuroscience.

[CR41] Luck SJ, Gaspelin N, Folk CL, Remington RW, Theeuwes J (2021). Progress toward resolving the attentional capture debate. Visual Cognition.

[CR42] Luck SJ, Hillyard SA (1994). Spatial filtering during visual search: Evidence from human electrophysiology. Journal of Experimental Psychology: Human Perception and Performance.

[CR43] Luck SJ, Thomas SJ (1999). What variety of attention is automatically captured by peripheral cues?. Perception & Psychophysics.

[CR44] Moher J, Egeth HE (2012). The ignoring paradox: Cueing distractor features leads first to selection, then to inhibition of to-be-ignored items. Attention, Perception, & Psychophysics.

[CR45] Müller HJ, Geyer T, Zehetleitner M, Krummenacher J (2009). Attentional capture by salient color singleton distractors is modulated by top-down dimensional set. Journal of Experimental Psychology: Human Perception and Performance.

[CR46] Ruthruff E, Gaspelin N (2018). Immunity to attentional capture at ignored locations. Attention, Perception, & Psychophysics.

[CR47] Sawaki R, Luck SJ (2010). Capture versus suppression of attention by salient singletons: Electrophysiological evidence for an automatic attend-to-me signal. Attention, Perception, & Psychophysics.

[CR48] Schneider, W. X. (2013). Selective visual processing across competition episodes: A theory of task-driven visual attention and working memory. *Philosophical Transactions of the Royal Society B: Biological Sciences*, *368*(1628). 10.1098/rstb.2013.0060 10.1098/rstb.2013.0060 PMC3758203 24018722

[CR49] Schönhammer JG, Becker SI, Kerzel D (2017). Which kind of attention is captured by cues with the relative target colour?. Visual Cognition.

[CR50] Shiu L-P, Pashler H (1994). Negligible effect of spatial precuing on identification of single digits. Journal of Experimental Psychology: Human Perception and Performance.

[CR51] Sperling G (1960). The information available in brief visual presentations. Psychological Monographs: General and Applied.

[CR52] Stilwell BT, Gaspelin N (2021). Attentional suppression of highly salient color singletons. Journal of Experimental Psychology: Human Perception and Performance.

[CR53] Theeuwes J (1991). Cross-dimensional perceptual selectivity. Perception & Psychophysics.

[CR54] Theeuwes J (1992). Perceptual selectivity for color and form. Perception & Psychophysics.

[CR55] Treisman A, Sato S (1990). Conjunction search revisited. Journal of Experimental Psychology: Human Perception and Performance.

[CR56] Treisman AM, Gelade G (1980). A feature-integration theory of attention. Cognitive Psychology.

[CR57] Wang B, Theeuwes J (2020). Salience determines attentional orienting in visual selection. Journal of Experimental Psychology: Human Perception and Performance.

[CR58] Weaver MD, van Zoest W, Hickey C (2017). A temporal dependency account of attentional inhibition in oculomotor control. NeuroImage.

[CR59] Wolfe, J. M. (2021). Guided Search 6.0: An updated model of visual search. *Psychonomic Bulletin & Review*, *28*(4), 1060–1092. 10.3758/s13423-020-01859-9 10.3758/s13423-020-01859-9 PMC8965574 33547630

