# Supplementary material for: Does attentional suppression occur at the level of perception or decision-making? Evidence from Gaspelin et al.’s (2015) probe letter task
Source: Psychol Res. 2022 Sep 12;87(4):1243–55. doi: 10.1007/s00426-022-01734-3 (PMC10191966; doi:10.1007/s00426-022-01734-3)
Supplement: Supplementary file 1 — Supplementary file1 (PDF 207 KB) [file 426_2022_1734_MOESM1_ESM.pdf]

Supplementary Material

Congruency effects in Experiment 3

In Experiment 3, two letters “o” and two letters “+” were randomly distributed across the four positions. As a result, the correspondence between the letter at the target position and the letter at the distractor or nontarget position could be evaluated. In previous research, it was observed that the identity of a colored distractor letter interfered with the discrimination of the target letter (Theeuwes & Burger, 1998; Zivony & Lamy, 2018). That is, responses were slowed by the presence of a colored distractor letter and the increase in reaction times (RTs) was more pronounced when the identity of the distractor did not correspond to the identity of the target letter (but see Becker, 2007). The effect of correspondence was attributed to “identity intrusions” from attended stimuli. In the probe task of the current paper, participants attended to the target of the search task, but were required to indicate the identity of the letter at distractor or nontarget locations. Because the target location was attended as part of the primary search task, we expect identity intrusions for trials where the letter at the distractor or nontarget locations had to be reported. To assess this hypothesis, we submitted percentages of correct responses to a 2 (shape: distractor, nontarget) x 2 (letter correspondence: corresponding, non-corresponding) repeated-measures ANOVA. Performance was better when the letter at the target location corresponded to the letter at the probed location (81.8% vs. 73.3%), $F(1, 35) = 25.87$, $p < .01$, $\eta_p^2 = .425$, confirming the expected identity intrusions from the probe letter at the target location. As in the analysis reported in the main text, there was no difference between distractor and nontarget shapes (76.5% vs. 78.6%), $F(1, 35) = 2.18$, $p = .14$, $\eta_p^2 = .059$. Importantly, there was no interaction between shape and letter correspondence, $F(1, 35) = 0.45$, $p = .51$, $\eta_p^2 = .013$, suggesting that effects of shape and correspondence were additive. Thus, the identity intrusions from the target shape were similar for reports of probe letters on distractor and nontarget shapes.

Effects of distractor repetition in Experiment 3

We asked whether the suppression effect would increase when a distractor-present trial was preceded by another distractor-present trial compared to when it was preceded by a distractor-absent trial. To assess effects of distractor repetition in the crucial comparison of

distractor and nontarget shapes, we submitted percentages correct to a 2 (shape: distractor, nontarget) x 2 (distractor on trial n-1: present, absent) repeated-measures ANOVA. As in the analysis reported in the main text, the difference between distractor and nontarget shapes was not reliable (77.7% vs. 80.4%), $F(1, 35) = 4.01$, $p = .053$, $\eta_p^2 = .103$. While the main effect of distractor presence on trial n-1 did not reach significance, $F(1, 35) = 0.69$, $p = .412$, $\eta_p^2 = .019$, there was a significant interaction, $F(1, 35) = 6.26$, $p = .02$, $\eta_p^2 = .152$. Suppression of the distractor relative to the nontarget shapes was more pronounced when the distractor was absent on trial n-1 (75.7% vs. 81.2%) compared to when it was present (79.7% vs. 79.5%). The interaction shows that distractor suppression did no increase over trials, but was strongest on the trial where it was triggered (i.e., after a distractor-absent trial).

However, the interaction of distractor presence and distractor repetition in the probe task was not matched by a corresponding interaction in RTs of the search task. A 2 (distractor: present, absent) x 2 (trial n-1: distractor-present, distractor-absent) repeated-measures ANOVA on RTs in Experiment 3 confirmed shorter RTs on distractor-present than -absent trials (700 vs. 711 ms), $F(1, 35) = 5.44$, $p = .03$, $\eta_p^2 = .134$, but neither the effect of distractor presence on trial n-1, $F(1, 35) = 0.85$, $p = .36$, $\eta_p^2 = .024$, nor the interaction, $F(1, 35) = 0.41$, $p = .53$, $\eta_p^2 = .012$, reached significance. Because results from the probe and search task did not match, further research is required to validate the idea that distractor suppression is weaker after repetition of the distractor.

Trial run length and effects of task switches in Experiment 3

We restricted the run length of the search task to 7 trials. As shown in supplementary Figure 1, this decreased the probability of a run length of 1-2 and increased the probability of run lengths between 3-7. As the goal of the probe task was to reflect the distribution of attention on search trials, these changes are desirable as very short runs are likely to disturb the search strategy and very long runs do not contribute to the probe task.

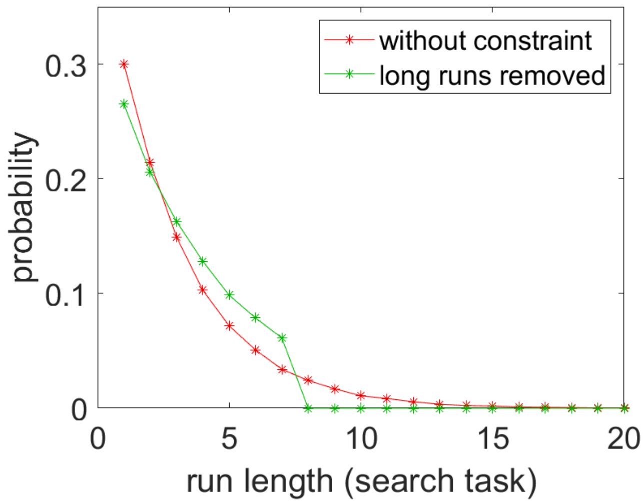

Supplementary Figure 1. Probability of different run lengths without constraint on run length and with runs longer than 7 removed. Probabilities were estimated by simulations with 1,000 iterations.

Because we had manipulated the distribution of task switches, we evaluated effects of task switches on the probe and search tasks. To assess effects of task switches on percentages correct in the crucial comparison of distractor and nontarget stimuli, we submitted percentages correct to a 2 (shape: distractor, nontarget) x 2 (task on trial n-1: same, different) repeated-measures ANOVA. Performance was better when the task on trial n-1 was the same compared to when it switched (84.4% vs. 77.5%), $F(1, 32) = 19.93$, $p < .01$, $\eta_p^2 = .363$. As in the analysis reported in the main text, there was no difference between distractor and nontarget locations (79.8% vs. 82.0%), $F(1, 35) = 2.47$, $p = .13$, $\eta_p^2 = .066$. Importantly, there was no interaction, $F(1, 35) < 0.01$, $p = .99$, $\eta_p^2 < .001$, suggesting that effects of shape and task on trial n-1 were additive.

In addition, we evaluated effects of task switches on RTs in the search task. To this end, we conducted a 2 (distractor: present, absent) x 2 (task on trial n-1: same, different) repeated-measures ANOVA on the RTs from the search task in Experiment 3. RTs were shorter on distractor-present than -absent trials (734 vs. 746 ms), $F(1, 35) = 5.34$, $p = .03$, $\eta_p^2 = .132$, mirroring the results in the main text. RTs were also shorter when the task on trial n-1 was the

same compared to when it was different (655 vs. 825 ms), $F(1, 35) = 122.61$, $p < .01$, $\eta_p^2 = .778$. However, the interaction was not significant, $F(1, 35) = 0.61$, $p = .44$, $\eta_p^2 = .017$, showing that task switching slowed responses, but did not affect the distribution of attention. Additive effects of task switching are consistent with a previous report showing that indicators of attentional capture in feature search did not change as a function of switch likelihood (Sali & Key, 2021).

References

Becker, S. I. (2007). Irrelevant singletons in pop-out search: Attentional capture or filtering costs? *Journal of Experimental Psychology: Human Perception and Performance, 33*(4), 764-787. https://doi.org/10.1037/0096-1523.33.4.764

Sali, A. W., & Key, J. (2021). Measuring attentional capture across learned states of cognitive flexibility. *Journal of Vision, 21*(9), 2875-2875. https://doi.org/10.1167/jov.21.9.2875

Theeuwes, J., & Burger, R. (1998). Attentional control during visual search: The effect of irrelevant singletons. *Journal of Experimental Psychology-Human Perception and Performance, 24*(5), 1342-1353. https://doi.org/Doi 10.1037//0096-1523.24.5.1342

Zivony, A., & Lamy, D. (2018). Contingent Attentional Engagement: Stimulus- and Goal-Driven Capture Have Qualitatively Different Consequences. *Psychological Science, 29*(12), 1930-1941. https://doi.org/10.1177/0956797618799302
